# Lipoprotein Glomerulopathy-Like Lesions in Atherosclerotic Mice Defected With HDL Receptor SR-B1

**DOI:** 10.3389/fcvm.2021.734824

**Published:** 2021-10-08

**Authors:** Jiawei Liao, Jie Bai, Xiangbo An, Yang Liu, Yuhui Wang, George Liu, Wei Huang, Yunlong Xia

**Affiliations:** ^1^Institute of Cardiovascular Diseases, First Affiliated Hospital of Dalian Medical University, Dalian, China; ^2^Institute of Cardiovascular Sciences and Key Laboratory of Molecular Cardiovascular Sciences, Ministry of Education, Peking University Health Science Center, Beijing, China; ^3^Department of Interventional Therapy, First Affiliated Hospital of Dalian Medical University, Dalian, China

**Keywords:** high-density lipoprotein, SR-B1, lipoprotein glomerulopathy, atherosclerosis, probucol

## Abstract

High-density lipoprotein (HDL) homeostasis is important in maintaining both cardiovascular and renal health. Scavenger receptor class B type 1 (SR-B1), the major HDL receptor in mammals, plays a crucial role in reverse cholesterol transport and HDL metabolism. Evidence from mouse study has well demonstrated that HDL disorders caused by Srb1 inactivation accelerate atherosclerosis and even induce lethal cardiovascular diseases. However, the renal consequences of Srb1 dysfunction are still unknown. Here we explored this issue in both Srb1 knockout (Srb1-/-) mice and atherosclerotic low-density lipoprotein receptor knockout (Ldlr-/-) mice with Srb1 deletion. Our data showed that no apparent renal damage was observed in 5-month-old Srb1-/- mice fed on standard rodent chow diet as well as Srb1-/- mice fed on a high-fat diet (HFD) for 12 weeks. However, 5-month-old Srb1/Ldlr-/- mice fed on rodent chow had increased urinary albumin excretion and developed spontaneous intraglomerular Oil-red O (ORO)-positive lipoprotein deposition that is similar to lesions observed in human lipoprotein glomerulopathy (LPG). HFD feeding accelerated LPG-like lesions in Srb1/Ldlr-/- mice, inducing severe proteinuria and significantly promoting intraglomerular ORO-positive lipoprotein deposition. Interestingly, probucol reversed HFD-induced HDL disorders and almost fully abrogated LPG-like lesions in Srb1/Ldlr-/- mice. In conclusion, the present study demonstrates that SR-B1 dysfunction leads to LPG-like lesions in atherosclerotic mice, which could be rescued by probucol. SR-B1 loss-of-function mutant carriers therefore might be susceptible to developing metabolic nephropathy in addition to cardiovascular diseases, and probucol might be a potential therapeutics.

## Introduction

High-density lipoproteins (HDLs) are traditionally known as cardio-protective lipoproteins for their crucial roles in reveres cholesterol transport (RCT) and possessing multiply anti-inflammatory, anti-oxidant and anti-thrombotic functions (1). In addition to their well-known cardiovascular benefits, there are increasing evidence suggesting that these lipoproteins are also closely associated with renal health and disease. Nephropathy, especially nephrotic syndrome or advanced chronic kidney diseases, could induce multiply alterations on the composition and function of HDLs, including reduced apolipoprotein A1 production and HDL-cholesterol (HDL-C) levels, accumulation of HDL unesterified cholesterol (UC) and triglycerides contents, reduced paraoxonase and enriched serum amyloid A that impair HDL-mediated RCT as well as anti-inflammatory and anti-oxidant capacity (2, 3). The formation of abnormal HDLs in renal diseases could then promote the onset and progression of cardiovascular complications, such as atherosclerosis, coronary heart disease, and stroke (4). Conversely, defective RCT and loss of HDL anti-inflammatory and anti-oxidant capacity due to HDL disorders might also lead to renal injuries. For examples, both clinical and experimental evidence has showed that defective HDLs caused by ATP-binding cassette transporter type A1 and lecithin–cholesteryl acyltransferase (LCAT) deficiencies are underlying contributory factors for renal diseases (5). Therefore, disruption of HDL metabolism is not only consequence of renal injuries but also responsible for the development and progression of renal diseases, which might in turn facilitate cardiovascular consequences.

Scavenger receptor class B type 1 (SR-B1) is the major HDL receptor in mammals (6, 7). Predominantly expressed in the liver and steroidogenic tissues, SR-B1 mediates the cellular uptake of HDL-C as well as peripheral cholesterol efflux to circulating HDLs, therefore crucial for RCT and HDL homeostasis (6, 7). Evidence from mouse study has shown that Srb1 dysfunction increases plasma HDL-C levels but impedes cholesterol esterification, leading to the production of UC-enriched dysfunctional “HDLs” (6, 7). These dysfunctional “HDLs” significantly promote atherosclerosis and even lead to coronary occlusion and lethal myocardial infarction in atherosclerotic apolipoprotein E (Apoe) deficient or low-density lipoprotein receptor knockout (Ldlr-/-) mice (8–11). Although the cardiovascular consequences of Srb1 deficiency have been well demonstrated, whether Srb1 dysfunction elicits renal damage is still unknown. Here we explored this issue in Srb1-/- mice and atherosclerotic Srb1/Ldlr-/- mice.

## Materials and Methods

### Animals and Diets

Mice (Srb1-/- mice and control wild-type mice; Srb1/Ldlr-/- and control Ldlr-/- mice) were generated and housed in ventilated cages as previously described (11). Diets used in the study included a standard rodent chow diet and a high-fat diet (HFD) (containing 0.5% cholesterol and 20% fat) supplemented with or without 1% probucol (Natural-Med, USA) (12). Only females were included in the study and all experimental procedures were in accordance with the Guide for the Care and Use of Laboratory Animals and approved by the Animal Care Committee at Dalian Medical University.

### Plasma Lipids

Blood was collected by retro-orbital bleeding after a 4-h fasting. Plasma total cholesterol (TC), HDL-C, and UC levels were determined by commercial kits (BioSino, China). Lipoprotein profiles were fractionated by a fast protein liquid chromatography (FPLC) system (Amersham Biosciences, UK) and measured as previously described (13).

### Renal Function and Histology

Urine samples were collected in metabolic cages (Tecniplast, USA). Urinary albumin concentration was determined with a Mouse Albumin ELISA Kit (Bethyl Laboratories, USA) and urinary albumin excretion (UAE) was represented by total albumin excretion in 24-h urine. Likewise, urinary creatinine (Ucr) concentration was determined with a creatinine parameter assay kit (R&D Systems, USA) and urinary creatinine excretion was represented by total creatinine excretion in 24-h urine. Renal samples used for histological analysis were collected at 5 months old on rodent chow diet and after 12 weeks on the HFD. Fixed paraffin sections were used for Periodic Acid-Schiff (PAS) staining, Mac2 (sc-20157, CST, USA) immunohistochemical staining and Masson staining, while frozen sections for Oil-red O (ORO) staining. Images were obtained and quantitated with Image J software.

### Statistics

All data were presented as mean ± SD. A Shapiro-Wilk normality test was first performed to determine whether the data were normally distributed. For two-group comparisons, normally distributed data were evaluated using unpaired *t*-test (parametric data) or Welch's *t* test (non-parametric data), while non-normally distributed data using Mann–Whitney test. For multiply-group comparisons, data were evaluated by two-way ANOVA. All evaluations were performed with Prism software and a *P* < 0.05 was regarded as significant.

## Results

### No Spontaneous Renal Damage in Srb1-/- Mice Fed on Standard Rodent Chow Diet

**Figure 1:**
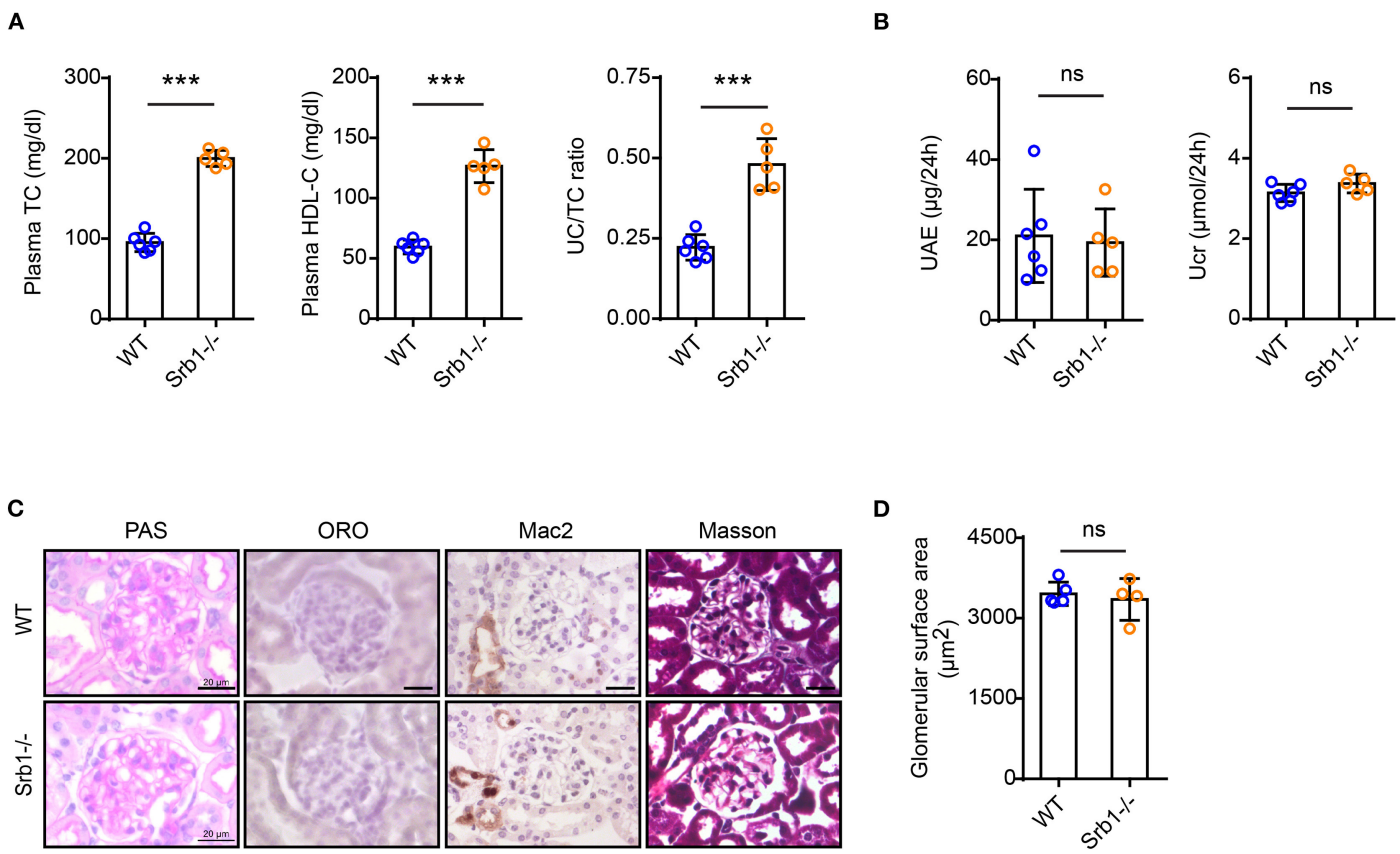
Plasma lipid parameters and renal function/histology in Srb1-/- and wild-type mice fed on standard chow diet. **(A)** Plasma TC levels, HDL-C levels, and UC/TC ratios; **(B)** 24-h UAE and Ucr; **(C)** Representative glomeruli stained with PAS, ORO, Mac2, and Masson; **(D)** Glomerular surface area. n = 3–6 per group. ***: *p* < 0.001; ns: no significance.

We first explored the renal phenotype in Srb1-/- mice fed on standard rodent chow diet. As shown in Figure 1A, plasma total cholesterol (TC), HDL-C and UC/TC ratio in Srb1-/- mice were all increased to twice of those in wild-type controls. However, when mice reached 5 months old on this standard rodent chow diet feeding, no significant change of renal function, reflected by 24-h urinary albumin excretion (UAE) and urinary creatinine (Ucr) level (Figure 1B), as well as glomerular morphology or size visualized by Periodic Acid-Schiff (PAS) staining, was observed in Srb1-/- mice, comparing with those in matched wild-type controls (Figures 1C,D). Moreover, no intraglomerular lipid deposition visualized by Oil-red O (ORO) staining, no inflammatory macrophage infiltration visualized by Mac2 immunohistochemical staining and no glomerular fibrosis visualized by Masson staining were observed in 5-month-old Srb1-/- mice (Figure 1C).

### High-Fat Diet (HFD) Feeding Did Not Induce Renal Damage in Srb1-/- Mice

**Figure 2:**
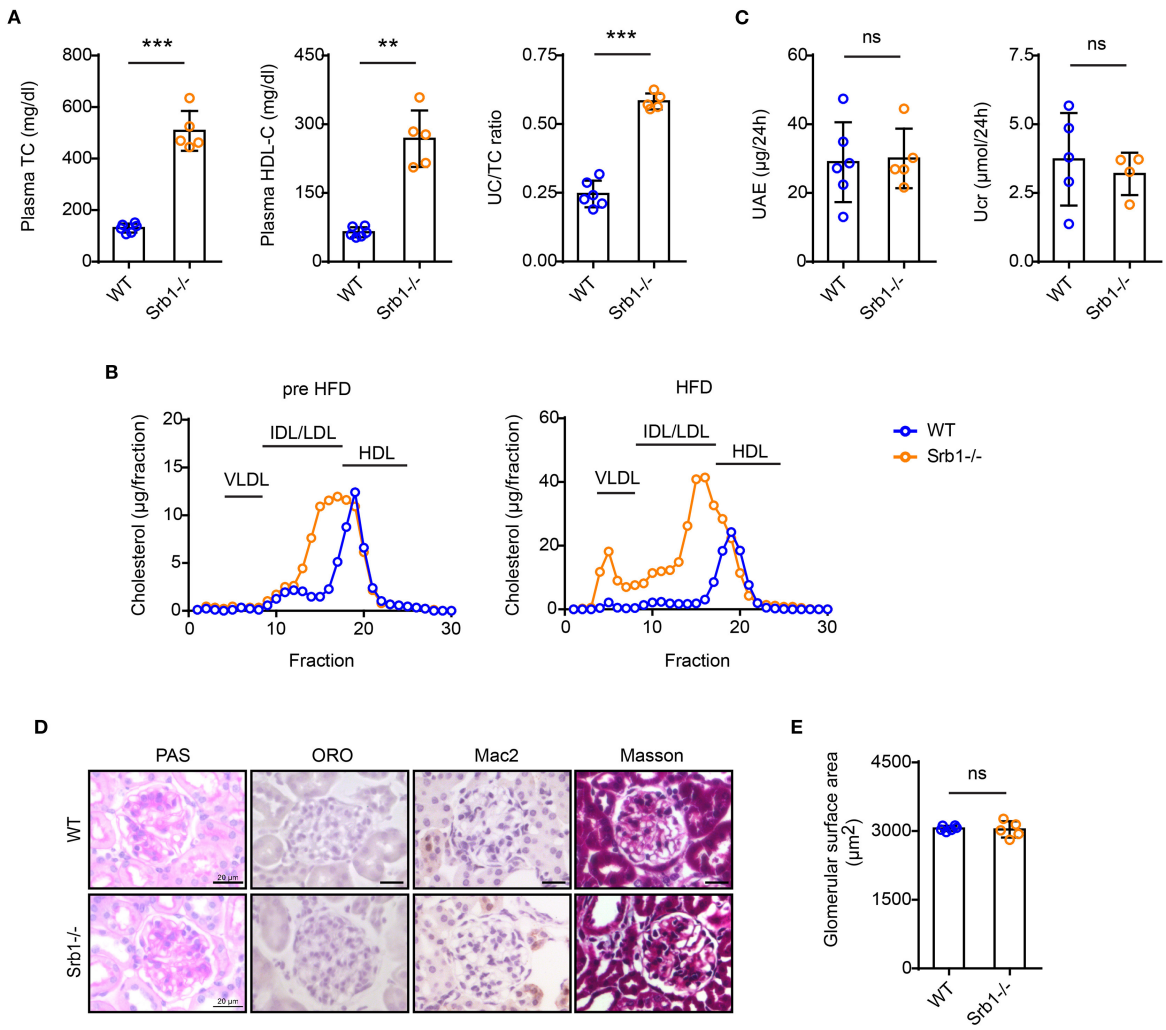
Plasma lipid parameters and renal function/histology in Srb1-/- and wild-type mice fed on HFD diet. **(A)** Plasma TC levels, HDL-C levels, and UC/TC ratios; **(B)** Lipoprotein profiles fraction by FPLC; **(C)** 24-h UAE and Ucr; **(D)** Representative glomeruli stained with PAS, ORO, Mac2, and Masson; **(E)** Glomerular surface area; **(E)** Quantitation of ORO-positive lipoprotein deposition. *n* = 5–6 per group. ^**^: *p* < 0.01; ^***^: *p* < 0.001; ns: no significance.

We then explored the effects of high-fat diet (HFD) feeding on the renal phenotype of Srb1-/- mice. Compared with a slightly increase of plasma lipid parameters in wild-type control mice, HFD feeding induced a further significant increase of plasma TC, HDL-C and UC/TC ratio in Srb1-/- mice (Figure 2A). Analysis of lipoprotein profiles by FPLC showed that upon HFD feeding, plasma cholesterol normally distributed in HDL fractions further increased and shifted toward LDL-sized fractions in Srb1-/- mice (Figure 2B). After 12 weeks on this HFD feeding, 24-h UAE and Ucr in Srb1-/- mice were found no significantly changed, comparing with those in wild-type controls (Figure 2C). Likewise, no significant change in glomerular morphology or size was observed in Srb1-/- mice (Figures 2D,E). Moreover, glomeruli in HFD-fed Srb1-/- mice had no intraglomerular lipid deposition, inflammatory macrophage infiltration or fibrosis (Figure 2D).

### Spontaneous Lipoprotein Glomerulopathy-Like Lesions in Srb1/Ldlr-/- Mice Even on Standard Rodent Chow Diet

**Figure 3:**
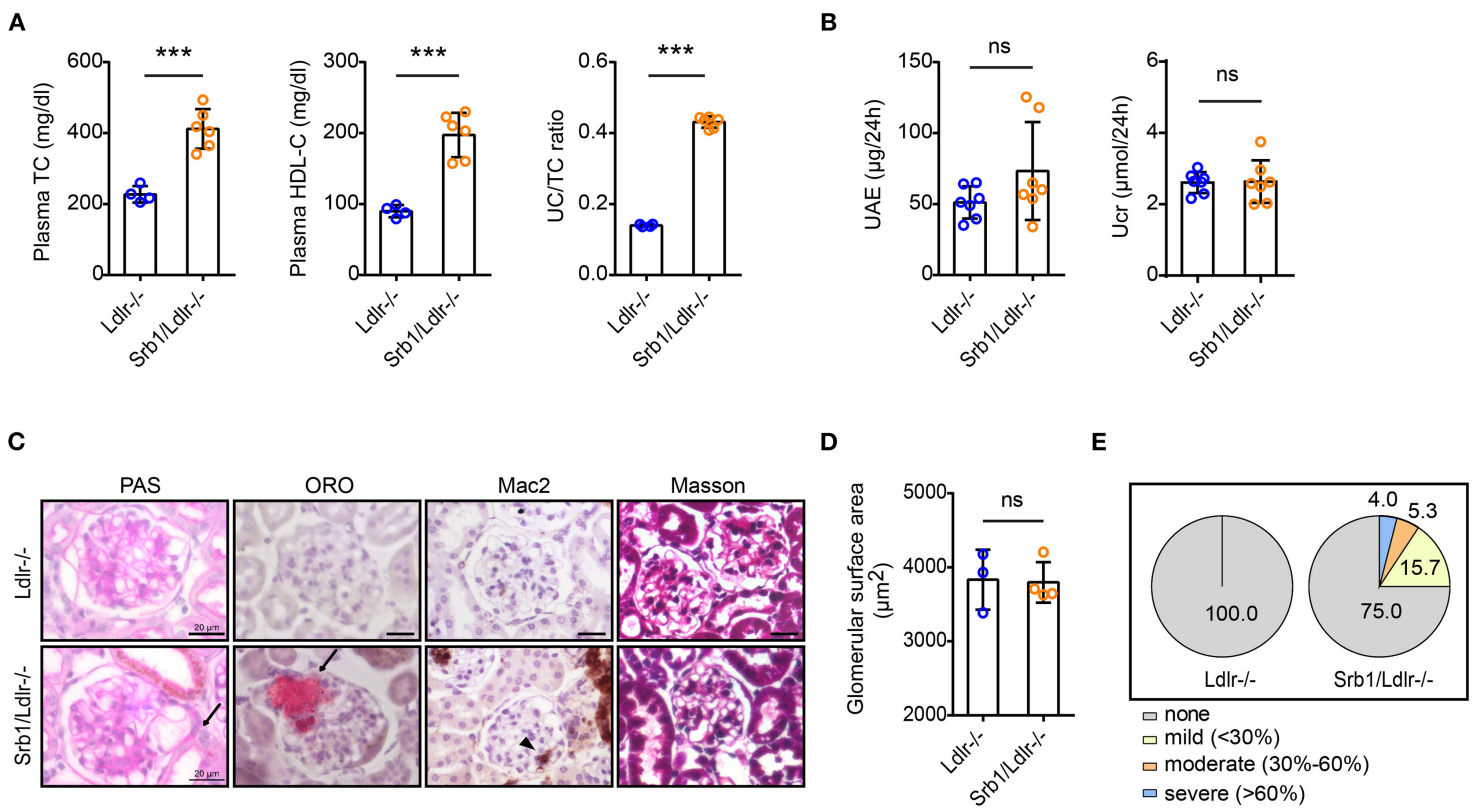
Plasma lipid parameters and renal function/histology in Srb1/Ldlr-/- and Ldlr-/- mice fed on standard chow diet. **(A)** Plasma TC levels, HDL-C levels, and UC/TC ratios; **(B)** 24-h UAE and Ucr; **(C)** Representative glomeruli stained with PAS, ORO, Mac2, and Masson. The arrows indicate foam-like lipoproteins. The triangle indicates macrophages; **(D)** Glomerular surface area; **(E)** Quantitation of ORO-positive lipoprotein deposition. *n* = 3–7 per group. ^***^: *p* < 0.001; ns: no significance.

We next explored the renal phenotype in atherosclerotic Srb1/Ldlr-/- mice. Similar to plasma parameter changes in Srb1-/- mice, plasma TC, and HDL-C as well as UC/TC ratio in chow-fed Srb1/Ldlr-/- mice were also twice of those in matched Ldlr-/- controls (Figure 3A). When mice reached 5 months of age on this rodent chow diet feeding, 24-h UAE in Srb1/Ldlr-/- mice increased slightly, while Ucr in Srb1/Ldlr-/- mice did not change, comparing with those in Ldlr-/- controls (Figure 3B). PAS staining showed that Srb1/Ldlr-/- mice developed intraglomerular foam-like lesions that were further identified as Oil-red O (ORO)-positive lipoprotein deposition reminiscent of lesions observed in human lipoprotein glomerulopathy (LPG), which were not seen in control Ldlr-/- mice (Figure 3C). To characterize the severity of intraglomerular lipoprotein deposition, we created a 4-degree criterion: none (no intraglomerular ORO-positive area), mild (intraglomerular ORO-positive area <30% of total glomerular surface area), moderate (intraglomerular ORO-positive area accounting for 30–60% of total glomerular surface area), and severe (intraglomerular ORO-positive area >60% of total glomerular surface area). According to this criterion, ~25% of total glomeruli in Srb1/Ldlr-/- mice developed intraglomerular ORO-positive lipoprotein deposition, of which 15% with mild deposition, ~6% moderate, and 4% severe deposition (Figure 3E). The deposition of ORO-positive lipoproteins in 5-month-old Srb1/Ldlr-/- mice did not lead to glomerular enlargement or fibrosis but attracted occasional macrophage infiltration (Figures 3C,D).

### High-Fat Diet (HFD) Feeding Significantly Aggravated LPG-Like Lesions in Srb1/Ldlr-/- Mice, Which Could Be Abrogated by Probucol Treatment

**Figure 4:**
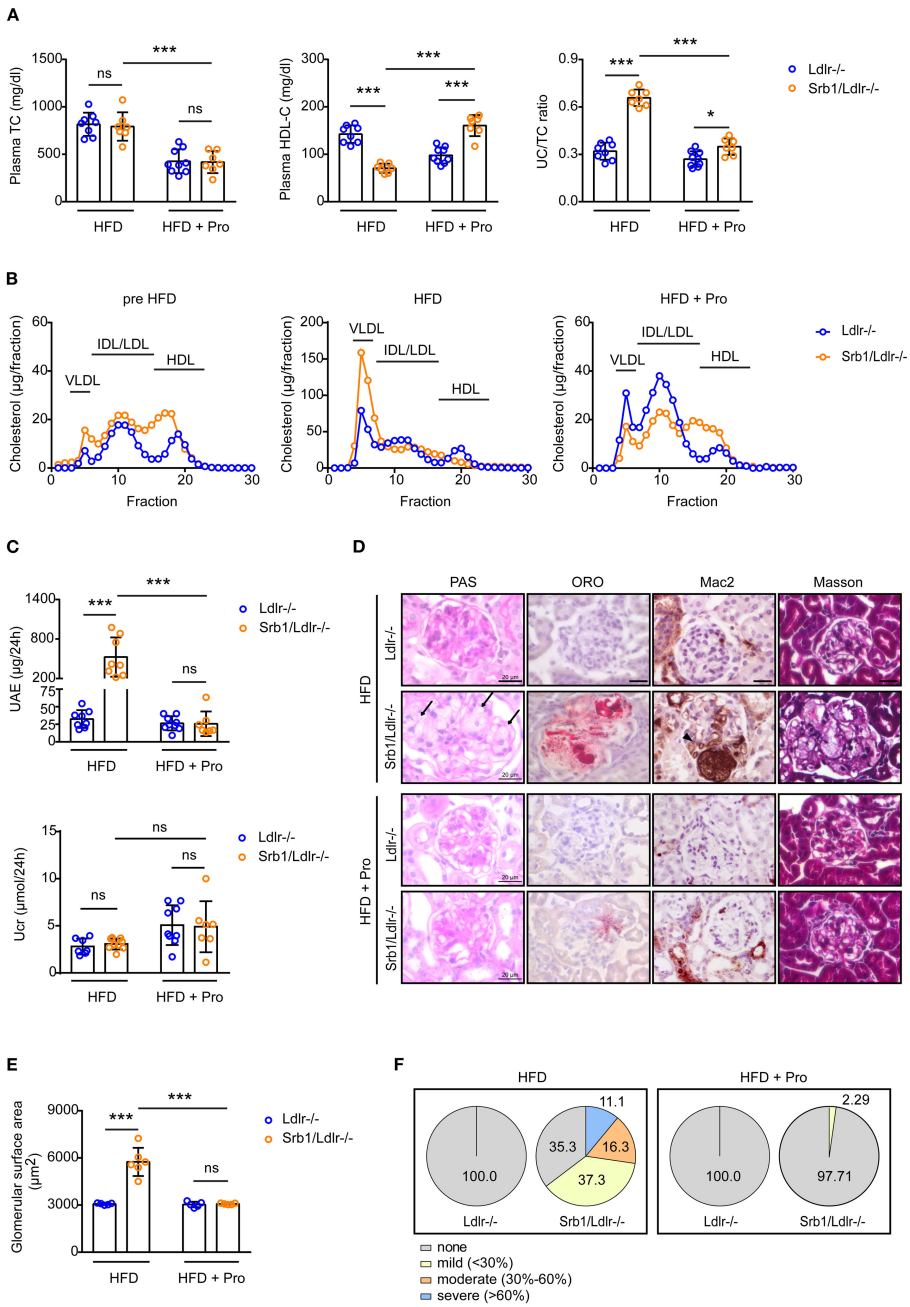
Plasma lipid parameters and renal function/histology in Srb1/Ldlr-/- and Ldlr-/- mice fed on HFD as well as HFD with probucol (Pro) supplement. **(A)** Plasma TC levels, HDL-C levels and UC/TC ratios; **(B)** Lipoprotein profiles fraction by FPLC; **(C)** 24-h UAE and Ucr; **(D)** Representative glomeruli stained with PAS, ORO, Mac2, and Masson. The arrows indicate foam-like lipoproteins. The triangle indicates macrophages; **(E)** Glomerular surface area; **(F)** Quantitation of ORO-positive lipoprotein deposition. *n* = 5–9 per group. ^*^: *p* < 0.05; ^***^: *p* < 0.001; ns: no significance.

Finally, we explored the effects of HFD feeding and probucol treatment on the LPG-like nephropathy. As we described earlier, this HFD feeding induces premature death in Srb1/Ldlr-/- mice (11). In fact, 1 of 7 Srb1/Ldlr-/- mice did not live to 12 weeks on the HFD feeding, therefore was excluded from this study. Analysis of those survived mice showed that both plasma TC level and UC/TC ratio in Srb1/Ldlr-/- mice and Ldlr-/- controls increased upon HFD feeding, although the increase of plasma TC in Srb1/Ldlr-/- mice were not as dominant as that in Ldlr-/- mice (Figure 4A). Unlike an increase of plasma HDL-C in Ldlr-/- mice, HFD induced a significant decrease of plasma HDL-C in Srb1/Ldlr-/- mice (Figure 4A). The diet-induced decrease of plasma HDL-C in Srb1/Ldlr-/- mice could be further confirmed by FPLC, which showed a significant cholesterol redistribution from HDL-sized lipoprotein particles to VLDL-sized particles (Figure 4B). Analysis into renal function showed that 24-h UAE in survived Srb1/Ldlr-/- mice increased more than 20-folds as compared with that in Ldlr-/- controls, suggesting a severe proteinuria in the mice (Figure 4C). In contrast, 24-h Ucr in survived Srb1/Ldlr-/- mice was found not significantly changed after HFD feeding (Figure 4C). Morphologically, HFD significantly increased glomerular size and ORO-positive lipoprotein deposition in Srb1/Ldlr-/- mice, while did not cause lipid accumulation in Ldlr-/- controls (Figures 4D,E). In fact, about 60% of total glomeruli in Srb1/Ldlr-/- mice were found deposited with ORO-positive lipoproteins, and those with moderate and severe lipoprotein deposition even reached 16 and 11% (Supplementary Figure 1 and Figure 4F). The deposited lipoproteins in the glomeruli of Srb1/Ldlr-/- mice might be cholesterol-dominant as shown by an increase of renal cholesterol content (Supplementary Figure 2) and led to severe intraglomerular inflammatory macrophage infiltration (Supplementary Figure 3 and Figure 4D) but not fibrosis (Figure 4D).

Probucol has been demonstrated to effectively correct HDL disorders and related metabolic diseases caused by Srb1 inactivation, including hemolysis (12), adipose loss (14), increased atherosclerosis and early-onset myocardial infarction (15) etc. Here we found that probucol treatment almost reversed plasma lipid parameters and lipoprotein profiles of HFD-fed Srb1/Ldlr-/- mice to the extent of chow-fed Srb1/Ldlr-/- mice (Figures 4A,B); furthermore, probucol treatment also rescued HFD-induced proteinuria (Figure 4C) and almost abrogated glomerular enlargement, intraglomerular lipoprotein deposition as well as macrophage infiltration in Srb1/Ldlr-/- mice (Figures 4D–F).

## Discussion

Wild-type mice are naturally resistant to atherosclerosis, probably because of their HDL-dominant athero-protective lipid profiles. Therefore, study of metabolic atherosclerotic diseases in mice are mostly performed in the context of Apoe or Ldlr defects (16). Previously, we have demonstrated that loss of Srb1 leads to accelerated atherosclerosis, coronary occlusion and lethal myocardial infarction in Ldlr-/- mice (11, 17). In the present study, we focused on the renal consequences. Our data showed that Srb1/Ldlr-/- mice but not Srb1-/- mice developed spontaneous and diet-aggravated intraglomerular lipoprotein deposition that is similar to lesions observed in human LPG. It seemed that atherosclerotic dyslipidemia was indispensable for the development and progression of the LPG-like nephropathy, as such lesions were not detected in Srb1-/- mice even on HFD feeding and could be reversed after correction of atherosclerotic dyslipidemia by probucol. Although data supporting this study only included those from females, we did observe that male Srb1/Ldlr-/- mice developed similar renal phenotypes (Supplementary Figure 4). Moreover, similar severe LPG-like lesions were also observed in chow-fed Srb1/Apoe-/- mice that died early from spontaneous acute coronary syndrome (Supplementary Figure 5). As renal diseases are closely related with cardiovascular health, our data raised new possibility that LPG-like nephropathy might be an important contributory factor for the onset and progression of atherosclerotic cardiovascular disease and premature death in Srb1/Apoe-/- and Srb1/Ldlr-/- mice, which were neglected previously. Meanwhile, Srb1/Apoe-/-, and Srb1/Ldlr-/- mice might be useful animal models for studying cardio-renal interactions.

Human LPG is a rare hereditary renal disease that is characterized by proteinuria and intraglomerular lipoprotein thrombi (18). Although the exact mechanism behind human LPG is still underdefined, Apoe deficiency is thought to be the major culprit, as several Apoe mutant carriers have been diagnosed with LPG and more causative relationship between Apoe abnormality and LPG could be found in mice (19, 20). For examples, glomeruli of aged Apoe-/-, and Apoe/Ldlr-/- mice are found with spontaneous lipoprotein thrombus deposition (21, 22) and expression of Apoe mutant could successfully induce LPG in Apoe-/- mice (23–25). In addition to Apoe defects, it seems that an abnormal lipoprotein called lipoprotein-X (Lp-X), which is enriched in phospholipid and UC but devoid of triglycerides and apolipoproteins, could serve as another underlying factor for LPG (26, 27). This abnormal lipoprotein usually occurs in LCAT deficiency and severe cholestasis (27). Interestingly, LCAT is the key enzyme for plasma UC esterification, whose deficiency leads to increased plasma UC/TC ratio (28). Furthermore, previous literatures have indicated that the atherosclerotic dyslipidemia in chow-fed Srb1/Apoe-/- mice is featured by the transformation of HDLs into extremely enlarged (VLDL-and LDL-sized) and UC-enriched particles that structurally resembles Lp-Xs (8, 15). In light of the significantly increased plasma UC/TC ratio and diet-induced transformation of abnormal VLDL-sized lipoprotein particles in Srb1/Ldlr-/- mice, it is reasonable to hypothesize that impeded LCAT activity and presence of Lp-Xs might be the underlying mechanism for the atherosclerotic dyslipidemia and LPG-like nephropathy in Srb1/Ldlr-/- mice, which however need to be defined in future.

Currently, several SR-B1 loss-of-function variants have been confirmed in human genetic studies, and most of them are indicated to increase cardiovascular risks (29–31). Unveiling the renal consequences of Srb1 deficient mice is therefore of novel clinical significance, as these variant carriers might also have increased risks of developing metabolic nephropathy, in addition to atherosclerotic cardiovascular diseases. Moreover, we provide experimental evidence that probucol is protective against SR-B1-associated renal diseases and might be a potential therapeutics for these mutant carriers, although the mechanism by which probucol rescues the atherosclerotic dyslipidemia and LPG-like nephropathy is still uncertain. As both increased plasma UC/TC ratio and diet-induced transformation of abnormal VLDL-sized lipoprotein particles in Srb1/Ldlr-/- mice were corrected after probucol treatment, we hypothesized that the therapeutic effects of probucol might be related with protection against LCAT inactivity and inhibition of Lp-X formation, which however also need to be defined further.

In conclusion, we demonstrate that SR-B1 dysfunction leads to LPG-like lesions in atherosclerotic mice, which could be reversed by probucol. Our study indicates that SR-B1 loss-of-function mutant carriers might be susceptible to developing metabolic nephropathy in addition to cardiovascular diseases, and probucol might be a potential therapeutics.

## Data Availability Statement

The original contributions presented in the study are included in the article/Supplementary Material, further inquiries can be directed to the corresponding author/s.

## Ethics Statement

The animal study was reviewed and approved by the Animal Care and Use Committee of Dalian Medical University.

## Author Contributions

JL: conceptualization, investigation, data curation, visualization, funding acquisition, and roles/writing—original draft. JB: investigation, data curation, and visualization. XA and YL: investigation. YW: methodology and data curation. GL: conceptualization and supervision. WH: conceptualization, funding acquisition, supervision, writing—review, and editing. YX: conceptualization, supervision, writing—review, and editing. All authors listed have made a substantial, direct and intellectual contribution to the work, and approved it for publication.

## Funding

Natural Science Foundation of Liaoning Province (2020-MS-270) to JL and National Natural Science Foundation of China (81770448) to WH.

## Conflict of Interest

The authors declare that the research was conducted in the absence of any commercial or financial relationships that could be construed as a potential conflict of interest.

## Publisher's Note

All claims expressed in this article are solely those of the authors and do not necessarily represent those of their affiliated organizations, or those of the publisher, the editors and the reviewers. Any product that may be evaluated in this article, or claim that may be made by its manufacturer, is not guaranteed or endorsed by the publisher.

## Supplementary Material

Supplementary Figure 1 Representative ORO-stained glomeruli of HFD-fed Srb1/Ldlr-/- mice.

Supplementary Figure 2 Quantification of renal cholesterol (left) and triglycerides (right) content in Srb1/Ldlr-/- and Ldlr-/- mice fed on HFD. n = 4 per group. ^**^: p < 0.01; ns: no significance.

Supplementary Figure 3 Representative Mac2-stained glomeruli of HFD-fed Srb1/Ldlr-/- mice and Ldlr-/- controls. The triangles indicate intraglomerular Mac2-positive macrophage clusters.

Supplementary Figure 4 Representative H&E-stained glomeruli of HFD-fed male Srb1/Ldlr-/- mice. The triangles indicate LPG-like lesions.

Supplementary Figure 5 Representative ORO-stained glomeruli of chow-fed Srb1/Apoe-/- mice died prematurely from spontaneous myocardial infarctions. The lower image was a magnified view of the black box of the upper image.

### Quantitation of Renal Lipid Content

Extraction and measurement of tissue lipids were performed as we previously described (13). In brief, kidney (approximately 100 mg) was weighed and homogenized in 1 mL PBS. Renal lipids were extracted using Folch's reagents (32) and dissolved in 1 mL 3% Triton X-100. Cholesterol and triglycerides contents in the solutions were determined by commercial enzymatic kits (Sigma, USA) and then normalized to renal weight.

## Abbreviations

HDL: high-density lipoprotein
RCT: reverse cholesterol transport
LCAT: lecithin-cholesterol acyltransferase
SR-B1: scavenger receptor class B type 1
HDL-C: HDL-cholesterol
UC: unesterified cholesterol
Apoe: apolipoprotein E
Ldlr: low-density lipoprotein receptor
FPLC: fast protein liquid chromatography
UAE: urinary albumin excretion
Ucr: urinary creatinine
PAS: periodic acid-schiff
ORO: Oil-red O
HFD: high-fat diet
Lp-X: lipoprotein-X.
