# Supplementary figures and images for: Lipoprotein Glomerulopathy-Like Lesions in Atherosclerotic Mice Defected With HDL Receptor SR-B1
Source: Front Cardiovasc Med. 2021 Oct 8;8:734824. doi: 10.3389/fcvm.2021.734824 (PMC8531488; doi:10.3389/fcvm.2021.734824)

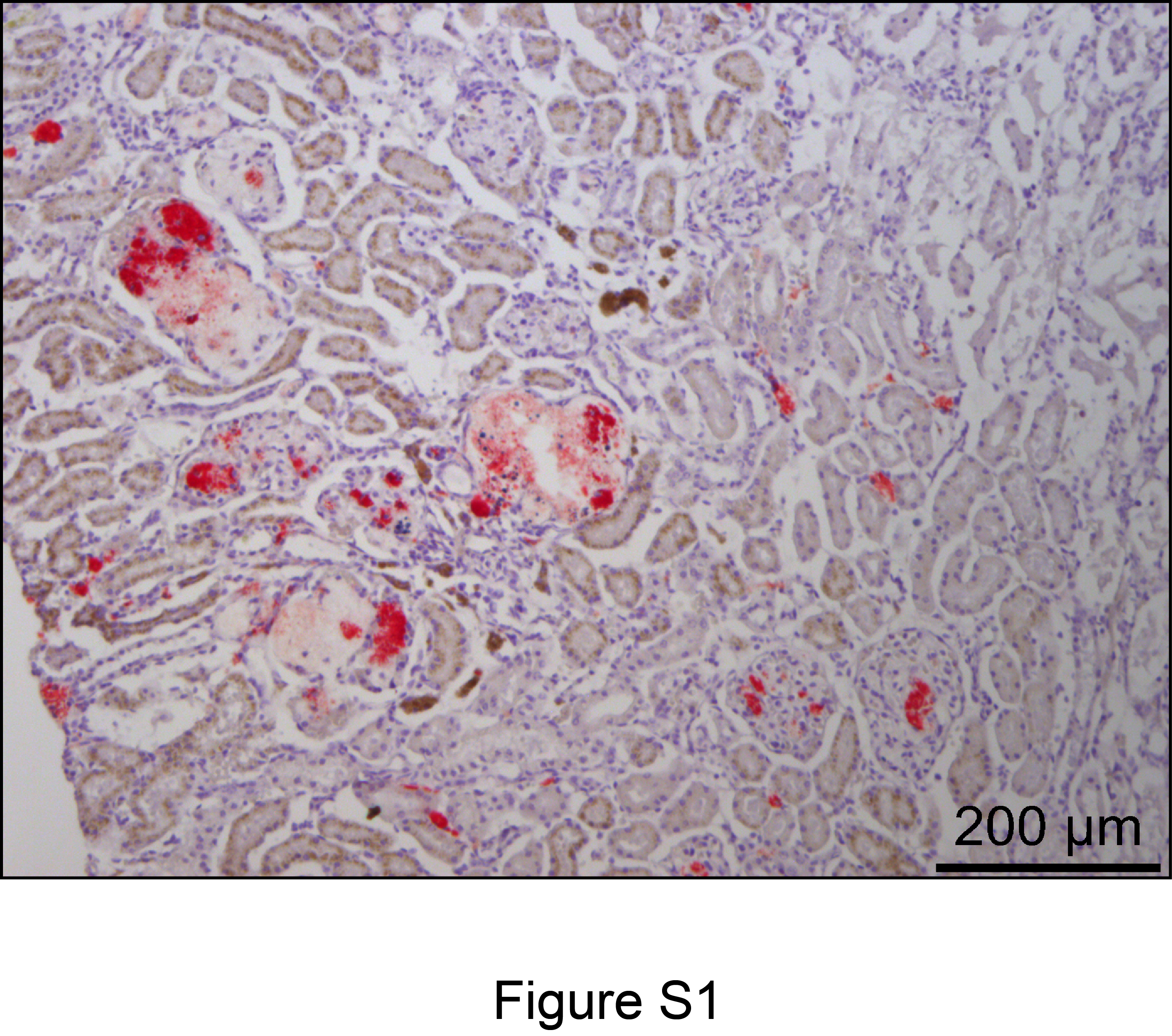

Supplement: Supplementary Figure 1 — Representative ORO-stained glomeruli of HFD-fed Srb1/Ldlr-/- mice. [file Image_1.JPEG]

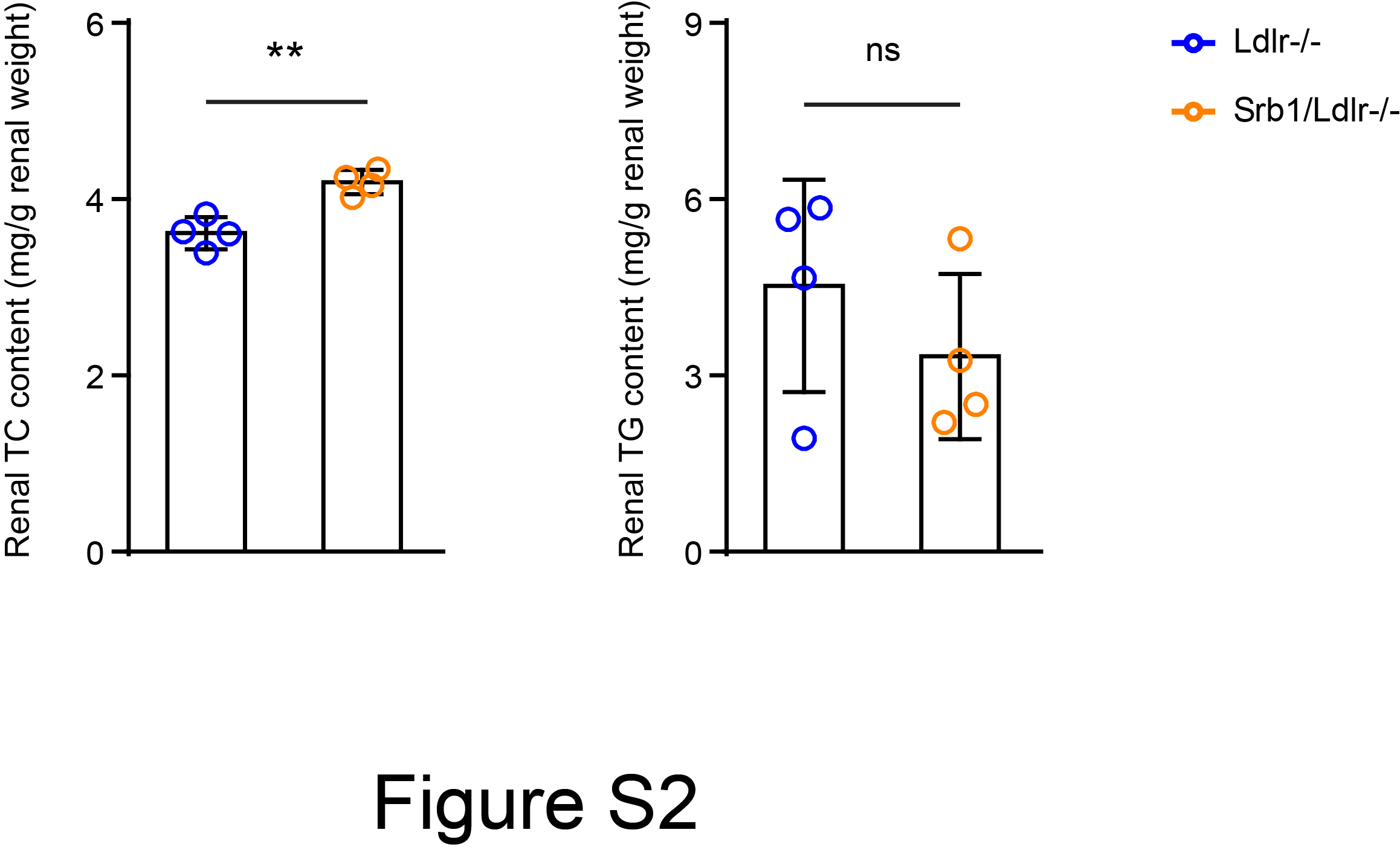

Supplement: Supplementary Figure 2 — Quantification of renal cholesterol (left) and triglycerides (right) content in Srb1/Ldlr-/- and Ldlr-/- mice fed on HFD. n = 4 per group. **: p < 0.01; ns: no significance. [file Image_2.JPEG]

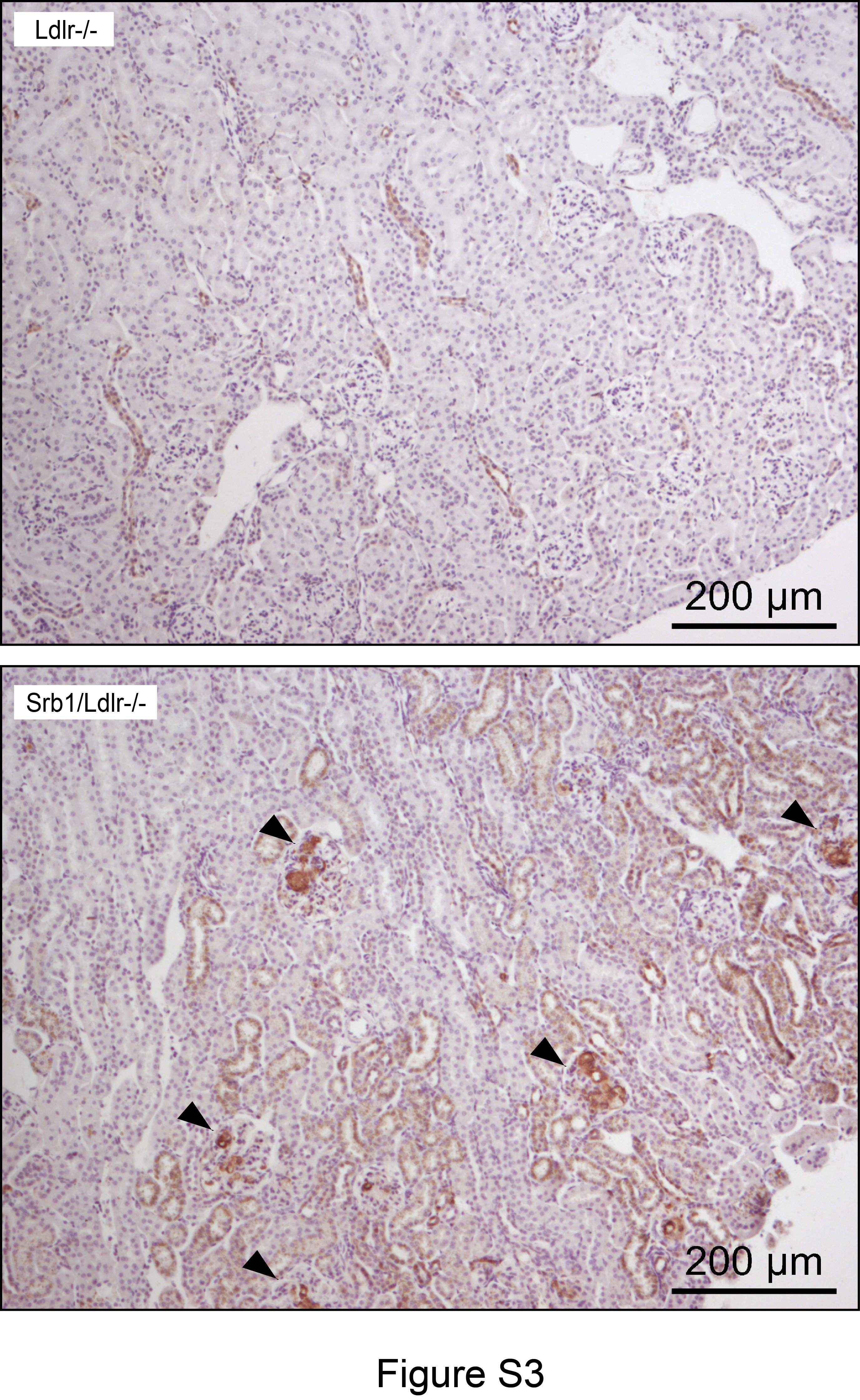

Supplement: Supplementary Figure 3 — Representative Mac2-stained glomeruli of HFD-fed Srb1/Ldlr-/- mice and Ldlr-/- controls. The triangles indicate intraglomerular Mac2-positive macrophage clusters. [file Image_3.JPEG]

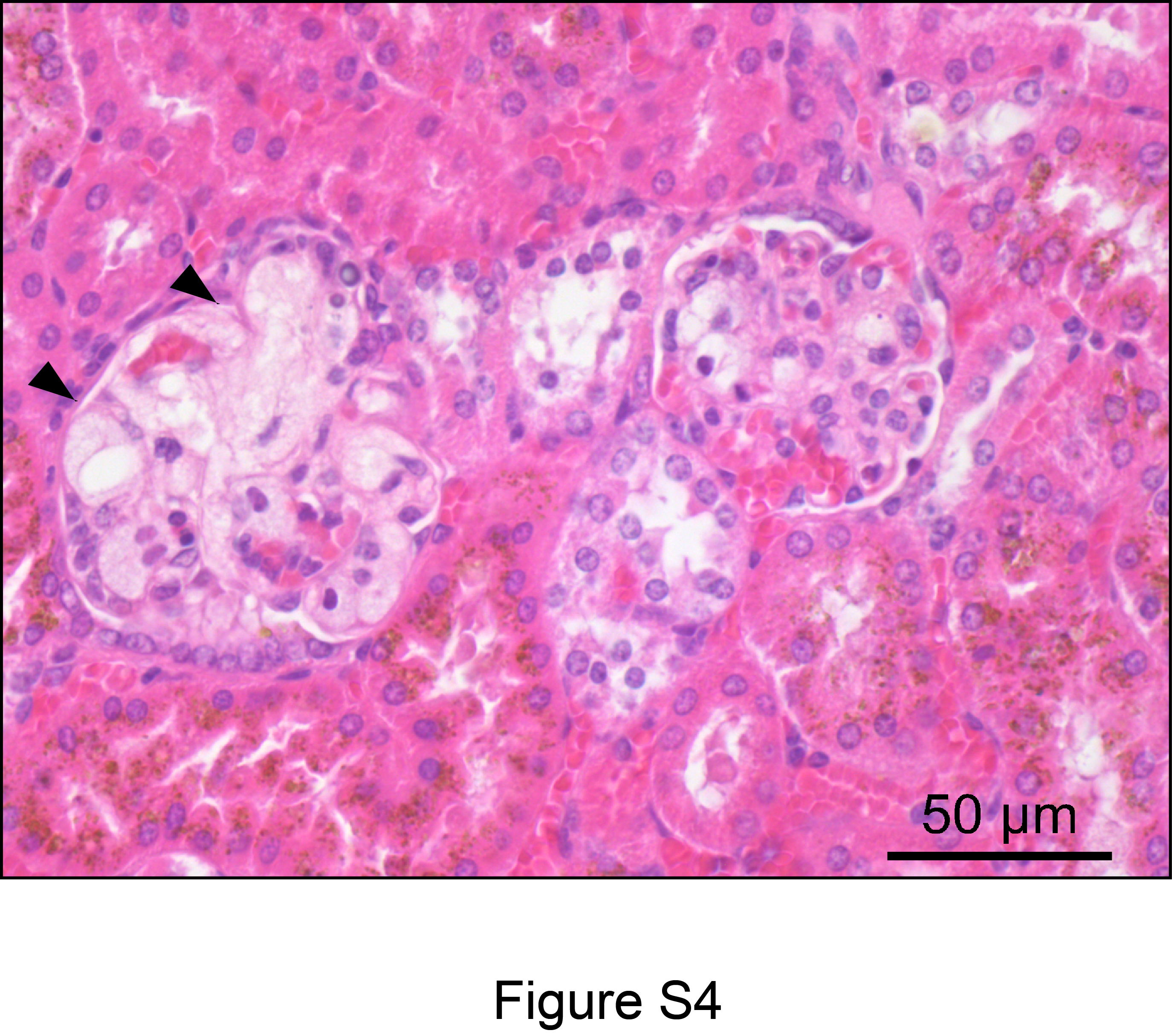

Supplement: Supplementary Figure 4 — Representative H&E-stained glomeruli of HFD-fed male Srb1/Ldlr-/- mice. The triangles indicate LPG-like lesions. [file Image_4.JPEG]

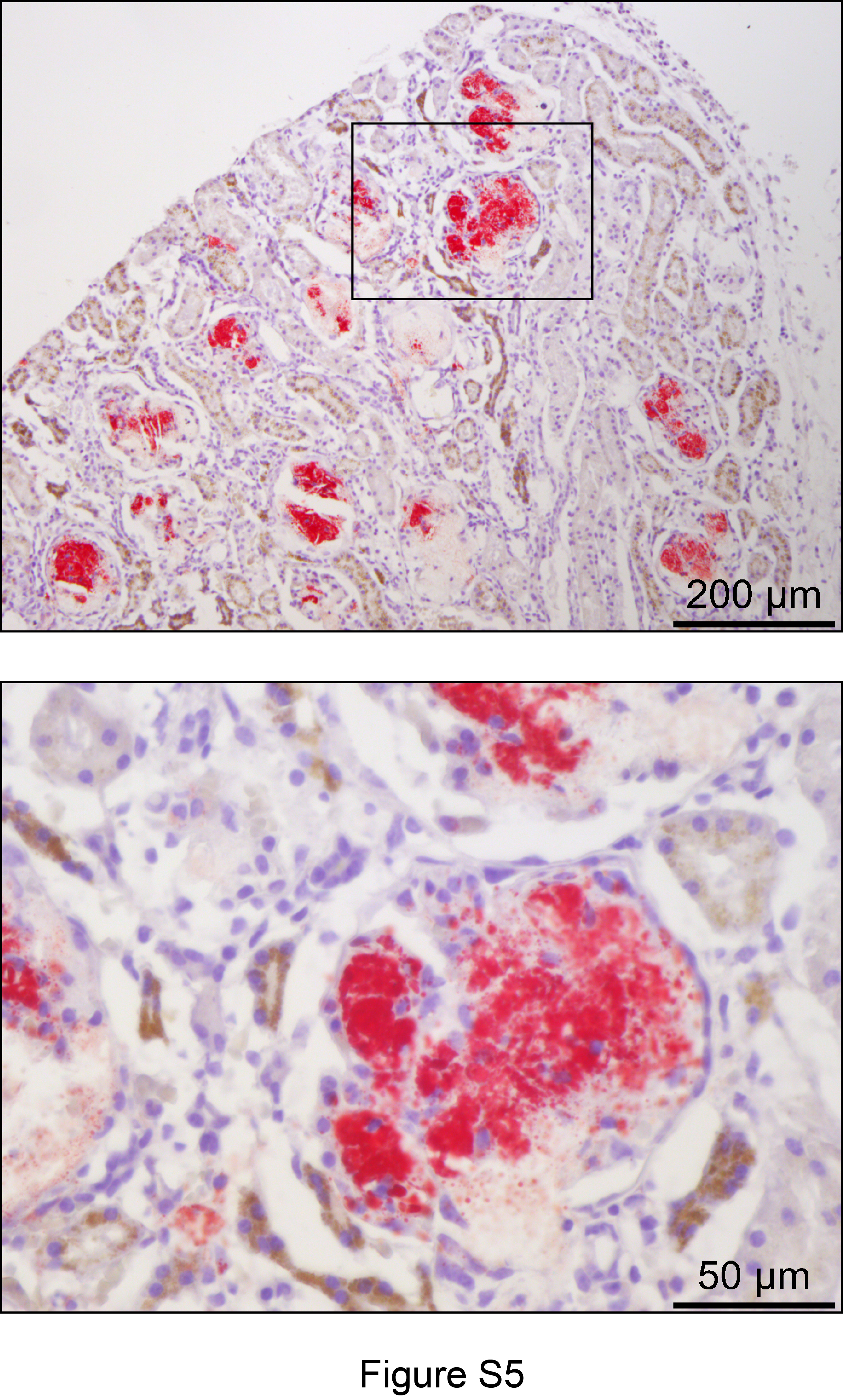

Supplement: Supplementary Figure 5 — Representative ORO-stained glomeruli of chow-fed Srb1/Apoe-/- mice died prematurely from spontaneous myocardial infarctions. The lower image was a magnified view of the black box of the upper image. [file Image_5.JPEG]
